# A Genome-Wide SNP Linkage Analysis Suggests a Susceptibility Locus on 6p21 for Ankylosing Spondylitis and Inflammatory Back Pain Trait

**DOI:** 10.1371/journal.pone.0166888

**Published:** 2016-12-14

**Authors:** Yanli Zhang, Zetao Liao, Qiujing Wei, Yunfeng Pan, Xinwei Wang, Shuangyan Cao, Zishi Guo, Yuqiong Wu, Ju Rong, Ou Jin, Manlong Xu, Zhiming Lin, Jieruo Gu

**Affiliations:** Department of Rheumatology and Immunology, The Third Affiliated Hospital of Sun Yat-Sen University, Guangzhou, Guangdong, P.R. China; Peking University First Hospital, CHINA

## Abstract

**Objectives:**

To screen susceptibility loci for ankylosing spondylitis (AS) using an affected-only linkage analysis based on high-density single nucleotide polymorphisms (SNPs) in a genome-wide manner.

**Patients and Methods:**

AS patients from ten families with Cantonese origin of China were enrolled in the study. Blood samples were genotyped using genomic DNA derived from peripheral blood leukocytes by Illumina HumanHap 610-Quad SNP Chip. Genotype data were generated using the Illumina BeadStudio 3.2 software. PLINK package was used to remove non-autosomal SNPs and to further eliminate markers of typing errors. An affected-only linkage analysis was carried out using both non-parametric and parametric linkage analyses, as implemented in MERLIN.

**Result:**

Seventy-eight AS patients (48 males and 30 females, mean age: 39±16 years) were enrolled in the study. The mean age of onset was 23±10 years and mean duration of disease was 16.7±12.2 years. Iritis (2/76, 2.86%), dactylitis (5/78, 6.41%), hip joint involvement (9/78, 11.54%), peripheral arthritis (22/78, 28.21%), inflammatory back pain (IBP) (69/78, 88.46%) and HLA-B27 positivity (70/78, 89.74%) were observed in these patients. Using non-parameter linkage analysis, we found one susceptibility locus for AS, IBP and HLA-B27 in 6p21 respectively, spanning about 13.5Mb, 20.9Mb and 21.2Mb, respectively No significant results were found in the other clinical trait groups including dactylitis, hip involved and arthritis. The identical susceptibility locus region spanning above 9.44Mb was detected in AS IBP and HLA-B27 by the parametric linkage analysis.

**Conclusion:**

Our genome-wide SNP linkage analysis in ten families with ankylosing spondylitis suggests a susceptibility locus on 6p21 in AS, which is a risk locus for IBP in AS patients.

## Introduction

Ankylosing spondylitis (AS) is a subtype of spondyloarthritis (SpA), a group of inflammatory axial and peripheral joint diseases. Although AS is a hereditary disease, some environmental factors have also been shown to be involved in the pathogenesis of the disease [1; 2]. In a twin-based study, the high concordance rate between monozygotic and dizygotic twins revealed that the heritability of AS exceeds 90% [3]. In addition, the recurrence risk for siblings has been reported to be as high as 82% [4]. In terms of genetic components, HLA-B27 is an important gene responsible for the familial aggregation of AS [5], with a concordance rate of 63% for monozygotic and 23% for dizygotic twin pairs [6]. HLA-B27 is considered to contribute about 20%-30% to the overall genetic risk for AS, while the whole MHC contributes approximately 40%-50% [3; 6], suggesting that other gene(s) might also play an important role in the development of the disease.

In recent years, genome-wide single nucleotide polymorphism (SNP) scan has become a popular approach for detecting new genetic variants in diseases including AS [7–9]. For example, in 2007, a genome-wide association study was applied to detect the genetic risk factors for AS with a custom-made Infinium array (14436 nsSNP) and showed that ARTS1 and IL-23R were susceptible genes besides HLA-B27 [9]. Apart from association analysis, linkage analysis is another way to detect susceptive gene(s) for genetic studies. Twelve loci have previously been confirmed to be associated with AS in Europeans (ANTXR2, CARD9, ERAP1, IL12B, IL23R, KIF21B, PTGER4, RUNX3, TBKBP1, TNFRSF1A and chromosomes 2p15 and 21q22) [8–10] and 2 other loci have recently been reported in Han Chinese (HAPLN1-EDIL3 and ANO6) [11].

A recent linkage analysis by microsatellites suggested that AS is an autosomal dominant disease in some large pedigrees with a penetrance of 0.56 in risk allele carriers. Accordingly, a susceptibility locus on chromosome 2q36.1-2q36.3 was identified later on [12]. Although dense SNP chip was used in association analysis, it is rarely adapted for linkage analysis in genetic disease, especially for AS. In addition, the susceptibility locus of AS was shown to be implicated in some genome-wide linkage studies as evaluated by microsatellite method [12–15]. Furthermore, the clinical traits of disease were rarely considered as a parameter for linkage analysis in AS and the risk factors for clinical traits of AS remained poorly understood. With linkage analysis, our study scanned AS cases in ten AS families using an Infinium array (Illumina) containing a panel of 620,901 SNPs, aiming to detect susceptible loci. Also, we conducted linkage analysis for the risk locis for five clinical traits (dactylitis, hip involvement, arthritis, IBP and HLA-B27) in those families.

## Materials and Methods

### Patients

Four hundred and one subjects from ten Chinese Han ethnic families were surveyed by experts in AS face to face. AS was diagnosed according to the modified New York criteria for AS [16]. Patients with other genetic diseases such as diabetes, nephropathy were excluded. All 78 AS patients from the families were enrolled in this study. The mean age of the patients was 39 ±16 years (ranging from 8 ~ 80 years). The study was approved by the Committee on Medical Ethics of the Third Affiliated Hospital of Sun Yat-sen University. All clinical investigations were conducted according to the principles expressed in the Declaration of Helsinki. Written informed consent was obtained from all the subjects. For the 5 subjects under the age of 18, written consent was obtained from their parents or legal guardians.

### Genotyping

Genomic DNAs were isolated from whole blood using a commercial DNA extraction kit (Qiagen). DNA was diluted to a concentration of 50 ng/ml (in 10mM Tris/1mM EDTA). Each sample was genotyped using approximately 200 ng of genomic DNA and Illumina HumanHap 610-Quad SNP Chip according to the recommended manufacture’s procedures (Illumina, US). The Illumina HumanHap 610-Quad beadchip consists of a panel of 620, 901 SNPs, among which 576, 112 are autosomal SNPs.

### Data formatting and quality control of genome-wide SNP linkage analysis

Genotype data were generated using Illumina BeadStudio 3.2 software. PLINK package [17]was used to remove non-autosomal SNPs and to further eliminate typing errors and uninformative markers. The exclusion criteria were minor allele frequency < 0.01, SNP call rate < 90%, and deviation from Hardy-Weinberg Equilibrium (p < 0.001). MERLIN program [18] was used to identify mistyped SNPs that are inconsistent with Mendelian inheritance. Moreover, to avoid bias in linkage calculation due to linkage disequilibrium (LD), one of each pair of SNPs was removed if the value of r^2^ is greater than 0.3. A total of 117 158 SNPs were included in linkage analysis ultimately.

### Linkage analysis

Firstly, for disease (AS) trait, we conducted an affected-only non-parametric and parametric linkage analysis in all 10 families. For parametric linkage analysis, MERLIN software was implemented [18; 19]. An autosomal dominant model was used, with an allele frequency of 0.003 and an assumed penetrance of 56% for the risk allele [12]. SNP genetic map positions were interpolated as their physical positions in megabyte. Then, linkage analysis was performed for other five clinical traits including dactylitis, hip involved, arthritis, IBP and HLA-B27.

### Statistical analysis

PLINK package was used for general statistical analysis. P value < 0.05 was considered to be statistically significant.

## Result

### 1. Clinical characteristics of patients

Among the 78 patients, the mean age of onset was 23±10 years and the mean duration of disease was 16.7±12.2 years. Iritis (2/76, 2.86%), dactylitis (5/78, 6.41%), hip joint involvement (9/78, 11.54%), peripheral arthritis (22/78, 28.21%), inflammatory back pain (69/78, 88.46%) and HLA-B27 positivity (70/78, 89.74%) were observed in these patients. (Table 1) Data was represented as mean ± SD.

**Table 1:** The clinical traits of 78 AS patients in ten families.

Families ID (case number)	Age (y)	Gendermale n(%)	Onset age (y)	Duration of disease (y)	Dactylitis n(%)	Hip involved n(%)	Arthritis n(%)	IBP n(%)	HLA-B27 n(%)
1 (n = 8)	32.00±12.97	6 (75.00)	22.00±8.21	9.75±8.50	3 (37.50)	0 (0)	5 (62.50)	8 (100)	8 (100)
2 (n = 8)	46.50±16.29	6 (75.00)	24.50±12.78	22.00±4.27	0 (0)	0 (0)	2 (25)	8 (100)	8 (100)
3 (n = 9)	30.67±16.04	5 (55.56)	19.22±8.40	11.47±9.72	0 (0)	4 (44.44)	2 (22.22)	6 (66.67)	9(100)
4 (n = 6)	39.00±16.55	3 (50.00)	28.50±15.71	10.58±10.76	0 (0)	1 (16.67)	0 (0)	2 (33.33)	3 (50.00)
5 (n = 7)	33.14±16.55	3 (42.86)	17.14±11.57	16.00±10.66	0 (0)	0 (0)	3 (42.86)	6 (85.71)	7(100)
6 (n = 10)	44.60±16.64	5 (50.00)	23.60±9.05	21.00±15.24	0 (0)	0 (0)	2 (20.00)	9 (90.00)	9 (90.00)
7 (n = 9)	45.22±7.24	5 (55.56)	23.56±8.57	21.44±11.80	0 (0)	2 (22.22)	5 (55.56)	9 (100)	8 (88.89)
8 (n = 8)	37.38±18.72	7 (87.50)	19.75±8.90	17.62±13.10	1 (12.50)	2 (25)	2 (25)	8 (100)	7 (87.50)
9 (n = 7)	44.14±17.14	3 (42.86)	26.00±6.22	18.14±19.51	1 (14.29)	0 (0)	1 (14.29)	7 (100)	5 (71.43)
10 (n = 6)	41.33±12.14	6 (100)	24.33±3.50	17.00±10.51	0 (0)	0 (0)	0 (0)	6 (100)	6 (100)

### 2. Identification of susceptibility locus for AS and other clinical traits by non-parameter linkage analysis

**Fig 1:**
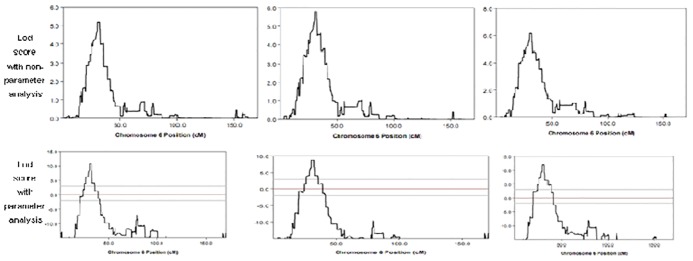
Lod score analysis for genome wide scan spanning the susceptibility region on Chromosome 6 for AS disease, IBP and HLA-B27 clinical traits.

**Fig 2:**
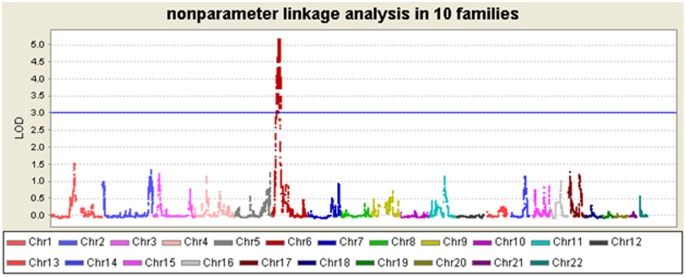
Lod score with non-parameter linkage analysis on all 22 chromosomes for AS disease for Illumina SNP markers.

Using non-parameter affected-only linkage analysis for AS disease, a susceptibility locus was found on 6p21, spanning a 13.5 Mb region (from 22296903 to 35831395) where the LOD scores were above 3 with the highest one reaching 5.164. No other locus was detected with LOD > 3 in other chromosomes (Figs 1 and 2). Using non-parameter linkage analysis for IBP trait, a susceptibility locus was detected on 6p21, spanning a 20.9Mb region (from 20334049 to 41191330) where the LOD scores were above 3 and the highest one reached 5.807. No other locus was detected with LOD > 3 in other chromosomes (Fig 1, Table 2).

**Table 2:** The peak lod score by linkage analysis for clinical traits of AS disease, IBP and HLA-B27 on 22 chromosomes.

Position	AS Disease (peak Lod score)		IBP (peak Lod score)		HLA-B27 (peak Lod score)	
	Non-parameter	Parameter	Non-parameter	Parameter	Non-parameter	Parameter
Chr1	1.51	-7.14	1.27	-9.95	1.39	-12.92
Chr2	1.33	-5.05	1.48	-8.03	1.45	-7.41
Chr3	1.24	-4.15	1.57	-7.88	0.59	-11.45
Chr4	1.16	-7.0	1.12	-11.41	0.89	-9.89
Chr5	1.22	-1.32	1.68	-6.93	1.70	-7.93
Chr6	5.16	10.76	5.80	8.77	6.14	11.96
Chr7	0.93	-5.78	0.95	-7.44	1.28	-11.17
Chr8	0.35	-7.83	0.50	-10.83	0.38	-10.68
Chr9	0.72	-4.76	0.87	-7.61	1.17	-9.85
Chr10	0.12	-7.86	0.32	-10.07	0.52	-10.05
Chr11	1.15	-2.95	1.62	-9.04	1.18	-8.43
Chr12	0.04	-8.49	0.12	-15.38	0.07	-13.63
Chr13	0.28	-9.98	0.32	-13.87	0.50	-13.26
Chr14	1.15	-1.53	1.30	-9.45	70	-13.49
Chr15	0.85	-4.01	1.05	-12.56	0.44	-12.62
Chr16	1.06	-5.80	0.69	-9.90	0.74	-11.37
Chr17	1.29	-4.51	1.66	-6.87	1.50	-10.99
Chr18	0.31	-11.22	0.22	-18.12	0.55	-16.38
Chr19	0.10	-8.09	0.13	-13.81	0.28	-14.34
Chr20	0	-8.37	0.01	-14.23	0.17	-14.52
Chr21	0.06	-6.47	0.05	-10.38	0.05	-10.96
Chr22	0.42	-5.22	0.82	-12.13	0.50	-12.44

A similar result was obtained in HLA-B27 trait by non-parameter linkage analysis. A susceptibility locus on 6p21 was identified, spanning a 21.2 Mb region (from 20334049 to 41550354) where the LOD scores were above 3 with the highest one reaching6.141. No other locus was detected with LOD score > 3 in other chromosomes (Fig 1, Table 2).

No locus with LOD score > 3 was detected in any chromosome in the non-parameter linkage analysis for other clinical trait groups including dactylitis, hip involved and arthritis.

### 3. Validation of the susceptibility locus on 6p21 by parametric linkage analysis

The above identified susceptibility loci on 6p21 were verified by a parametric linkage analysis using an autosomal dominant model, with a LOD score above 3 (Fig 1). Similar regions were detected by the parametric linkage analysis in the clinical traits of AS, IBP and HLA-B27, respectively. The same susceptibility locus among these three traits ranged from 26, 283, 831 to 35, 727, 532, spanning above 9.44 Mb region. The highest LOD scores were all less than 3 in the analysis of other clinical traits including dactylitis, hip involved and arthritis traits.

## Discussion

To the best of our knowledge, this study is the first one that used high-density single nucleotide polymorphisms (SNPs) to screen susceptibility loci for AS with an affected-only linkage analysis in ten AS families and validated the susceptibility locus with an association analysis between sporadic AS patients and healthy controls in the same array. In previous studies, microsatellite marker was the most common method for linkage analysis in the study of genetic diseases, especially in AS families [12; 13; 15]. Some studies have provided suggestive evidence for the presence and location of some non-MHC genes that influence the susceptibility to AS [2; 8]; nevertheless, the linkage between MHC and AS proven by microsatellite markers is the strongest [20; 21]. In our study, linkage analysis with a high-density SNPs array was used for genome-wide scan in AS families, and a susceptibility locus on 6p21 was identified with non-parameter and parameter analysis, which is the first strong evidence supporting the linkage between MHC and AS by SNPs markers besides microsatellite markers. Besides the susceptibility locus on 6q21, a new locus on the chromosome 6q21.3 was identified for familial chronic lymphocytic leukemia [22], nodular sclerosing Hodgkin lymphoma [23], and diffuse large B-cell lymphoma [24] by GWAS study.

Secondly, the significant heritability of IBP and HLA-B27 was identified in the current study. For the first time, we confirmed the risk locus for IBP in AS patients by linkage analysis, demonstrating that the susceptibility locus on 6p21 was linked to IBP trait in AS. However, the possible susceptibility gene(s) for IBP on 6p21 remains unknown and are needed to be further explored. Previous studies pointed out that IBP was a frequent clinical trait of AS [25]and manifestation of axial skeleton inflammation [26]. In other studies, TNF-alpha mRNA and protein were detected in dense cellular infiltrates of sacroiliac joint biopsy specimens from patients with AS, indicating that TNF-alpha could have implications on potential immunotherapeutic approaches to this disease[27]. Interestingly, TNF-alpha gene was found on 6p21. However, whether TNF-alpha was the susceptibility gene involved in the pathogenesis of AS remains unclear and requires further investigation. Complex (MHC) is located on chromosome 6 and presents antigenic peptides (derived from self and non-self antigens) to T cells. In our study, we demonstrated the susceptibility region was 6p21 when HLA-B27 was considered as a clinical trait of AS for linkage analysis. This finding is a proof of the accuracy of our linkage analysis result, because HLA-B27 is located on 6p21 in the gene map. We also showed that HLA-B27 was significantly linked with AS by a genome-wide linkage study in dense SNP array, which is consistent with several previous studies demonstrating the close association between HLA-B27 and AS [28; 29].

Furthermore, only a small proportion of B27-positive Caucasians develop AS [30], indicating that although B27 is almost essential for the inheritance of AS within families, there are other genetic risk factors involved. Recurrence risk ratio modelling in different degrees of relatives suggests that 2–6 genes are likely to interact multiplicatively in determining the susceptibility to AS [4]. In agreement with this, our study indicates that other possible susceptibility genes may be located on 6p21 that are narrowed by a LOD threshold above 8.4 in parametric linkage analysis. Nevertheless, due to the limited number of patients enrolled in the present study, large cohort clinical studies are required to confirm this finding in the future.

In conclusion, our genome-wide SNP linkage analysis in ten families with ankylosing spondylitis suggests a susceptibility locus on 6p21 for AS, which is a risk locus for IBP in AS patients.

## Supporting Information

S1 File websites and links for supported foundations. (DOCX)

S2 File regions found in the article. (DOCX)
