# Supplementary material for: A Genome-Wide SNP Linkage Analysis Suggests a Susceptibility Locus on 6p21 for Ankylosing Spondylitis and Inflammatory Back Pain Trait
Source: PLoS One. 2016 Dec 14;11(12):e0166888. doi: 10.1371/journal.pone.0166888 (PMC5156442; doi:10.1371/journal.pone.0166888)
Supplement: S1 File — (DOCX) [file pone.0166888.s001.docx]

National Natural Science Foundation for the youth NSFY of China (Grant No.81302583) : <https://isisn.nsfc.gov.cn/egrantweb/contract/index>


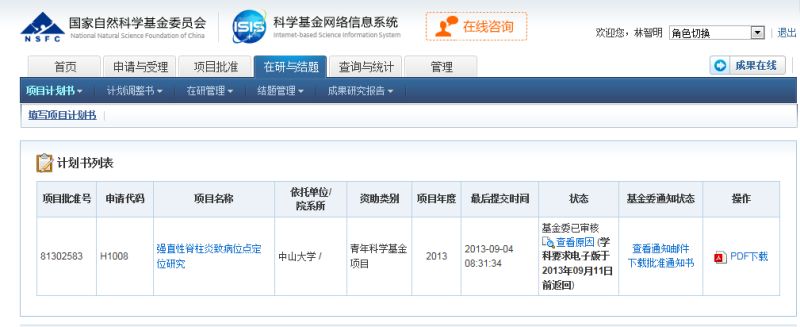


Guangdong Natural Science Funds for Distinguished Young Scholar (Grant No.2014A030306039) <http://pro.gdstc.gov.cn/egrantweb/prpapprove/lxxm>
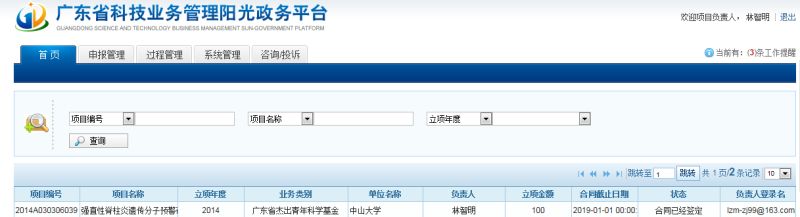


High-level personnel of special support program for Technology Innovative Talents and the Top Young of Guangdong Province（Grant No.2015TQ01R516）: [http://rc.gdstc.gov.cn/egrantweb/main###](http://rc.gdstc.gov.cn/egrantweb/main##)


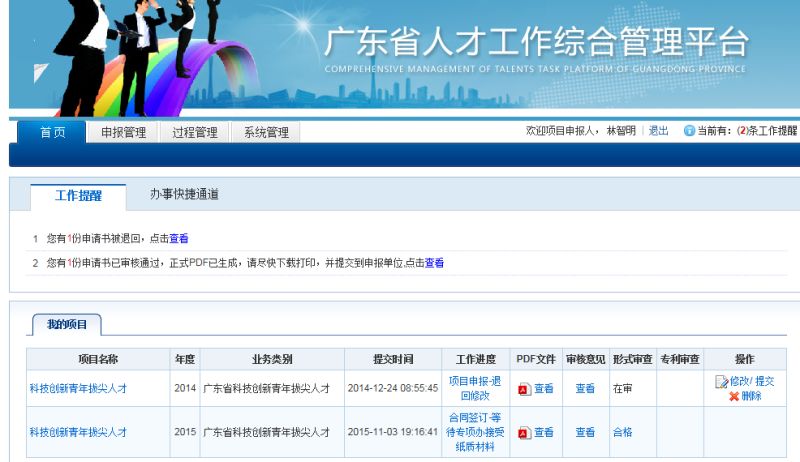


Pearl River Nova Program of Guangzhou(Grant No. 201610010005): <http://wsbs.gzsi.gov.cn/privateportal.htm>


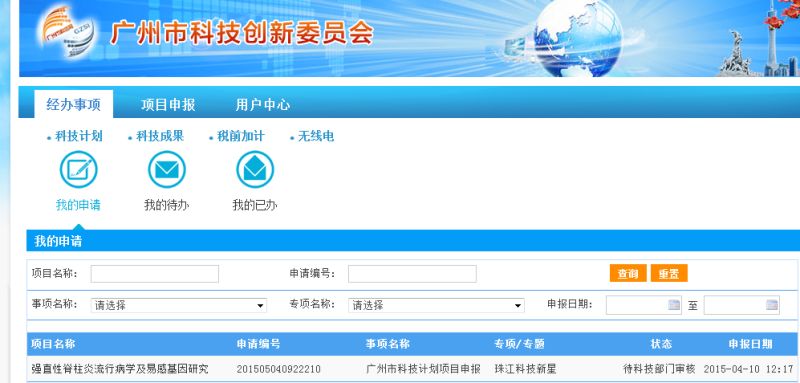


For the other three foundations from the university, there is no website to track.
