# Supplementary material for: A Genome-Wide SNP Linkage Analysis Suggests a Susceptibility Locus on 6p21 for Ankylosing Spondylitis and Inflammatory Back Pain Trait
Source: PLoS One. 2016 Dec 14;11(12):e0166888. doi: 10.1371/journal.pone.0166888 (PMC5156442; doi:10.1371/journal.pone.0166888)
Supplement: S2 File — (DOCX) [file pone.0166888.s002.docx]

**by non-parameter linkage analysis:**

AS: 13.5Mb region (from 22296903 to 35831395) : <http://genome.ucsc.edu/cgi-bin/hgTracks?db=hg38&lastVirtModeType=default&lastVirtModeExtraState=&virtModeType=default&virtMode=0&nonVirtPosition=&position=chr6%3A26808400-31319897&hgsid=551501585_VByTtfqxKNadye6WgNURgq9otSss>

IBP trait: 20.9Mb region (from 20334049 to 41191330) :

<http://genome.ucsc.edu/cgi-bin/hgTracks?db=hg38&lastVirtModeType=default&lastVirtModeExtraState=&virtModeType=default&virtMode=0&nonVirtPosition=&position=chr6%3A20334049-41191330&hgsid=551513531_A7ywaHaMOHCLsbEXze3r09ZW1MVy>

HLA-B27 trait: 21.2 Mb region (from 20334049 to 41550354): <http://genome.ucsc.edu/cgi-bin/hgTracks?hgsid=551526101_sOrcRVebMOPuBOJA5jJHKjLHXfPC&org=Human&db=hg38&position=chr6%3A20334049+-+41550354&pix=1243>

**by parametric linkage analysis:**

AS, IBP, HLA-B27: 9.44 Mb ( from 26,283,831 to 35,727,532) <http://genome.ucsc.edu/cgi-bin/hgTracks?hgsid=551522035_n7u4llvfNxG1cMIOXIiTInidG1GM&org=Human&db=hg38&position=chr6%3A+26%2C283%2C831+-+35%2C727%2C532&pix=1243>
